# Axillary Brachial Plexus Block

**DOI:** 10.1155/2011/173796

**Published:** 2011-05-22

**Authors:** Ashish R. Satapathy, David M. Coventry

**Affiliations:** Department of Anaesthesia, Ninewells Hospital and Medical School, Dundee DD1 9SY, UK

## Abstract

The axillary approach to brachial plexus blockade provides satisfactory anaesthesia for elbow, forearm, and hand surgery and also provides reliable cutaneous anaesthesia of the inner upper arm including the medial cutaneous nerve of arm and intercostobrachial nerve, areas often missed with other approaches. In addition, the axillary approach remains the safest of the four main options, as it does not risk blockade of the phrenic nerve, nor does it have the potential to cause pneumothorax, making it an ideal option for day case surgery. Historically, single-injection techniques have not provided reliable blockade in the musculocutaneous and radial nerve territories, but success rates have greatly improved with multiple-injection techniques whether using nerve stimulation or ultrasound guidance. Complete, reliable, rapid, and safe blockade of the arm is now achievable, and the paper summarizes the current position with particular reference to ultrasound guidance.

## 1. Introduction

The axillary approach to brachial plexus was first demonstrated in 1884 by William Halsted when he injected cocaine under direct vision [1]. In 1911, G. Hirschel performed the first percutaneous axillary block [2]. It was only after Burnham's publication in 1959 [3] that this block gained popularity among anaesthetists. Since then, it has become the most used peripheral nerve block for forearm and hand surgery, especially due the low incidence of complications compared to the more proximal approaches to the brachial plexus.

## 2. The Brachial Plexus in the Axilla [4]

The brachial plexus supplies the nerve supply to the upper limb and is formed by the ventral rami of the lower four cervical nerves and the first thoracic nerve. It consists of roots, trunks, divisions, and cords. The roots are arranged between the scalenus anterior and medius muscles, and they combine in the posterior triangle to form three trunks: upper, middle, and lower. On approaching the clavicle, each of the three trunks divides into an anterior and posterior division to supply the flexor and extensor compartments of the arm, respectively. Anterior divisions of the upper and middle trunk unite to form the lateral cord, anterior division of the lower trunk continues as the medial cord, and posterior divisions of all the three trunks assemble to from the posterior cord. The three cords enter the axilla at the apex and are arranged, according to the names, around the second and third parts of the axillary artery. In relation to the first part of the artery, however, the lateral and posterior cords are lateral, and the medial cord lies posterior to the artery.

**Figure 1:**
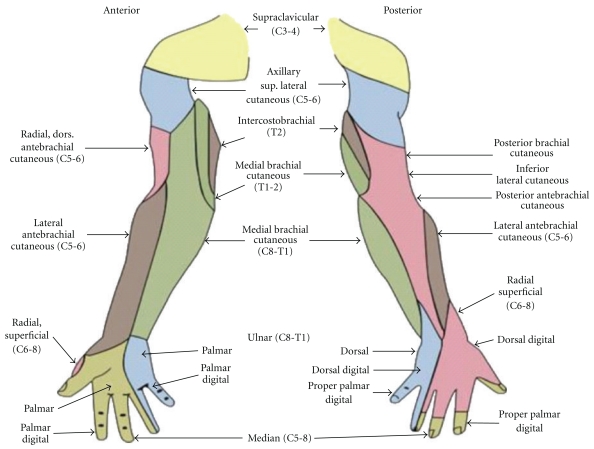
Cutaneous innervation of the upper extremity. Note the significant contributions of the cutaneous branches of the plexus. (Courtesy From Wikimedia Commons, file: Gray's Anatomy 812 and 814.PNG).

At the lateral border of the pectoralis minor muscle, the cords divide into terminal nerves of the brachial plexus: musculocutaneous, median, ulnar, radial, axillary, medial cutaneous nerve of arm (MCNA), and medial cutaneous nerve of forearm (MCNF), which along with the intercosto-brachial nerve (ICB) provide the sensory and motor supply to the whole upper extremity (Figure 1). The cords, the terminal branches, and the vessels lie within an incomplete fascial sheath derived from the scalene fascia, which is in turn derived from the prevertebral fascial layer. 

At the level of axilla, the median, radial, and ulnar nerves lie within the neurovascular bundle, whereas the median cutaneous nerve of the arm and forearm may lie either inside or outside the sheath. The musculocutaneous nerve always lies outside the sheath (in the plane between the biceps and coracobrachialis muscle or in the body of coracobrachialis), because it leaves the lateral cord before the cords enter the axilla. Within the fascia, in relation to the axillary artery, the nerves are arranged as follows: (1) median-lateral and anterior, (2) ulnar-medial and anterior, and (3) radial-medial and posterior. The musculocutaneous nerve appears lateral and posterior to the artery.

## 3. Basic Principles of Brachial Plexus Block

The best approach to brachial plexus is determined by the sensory and motor innervations of the surgical site concerned and the potential adverse effects of each. Hence, for shoulder and proximal humeral procedures, an interscalene block is performed, which reliably blocks C5-C6 nerve roots and proximal branches such as the suprascapular nerve. A supraclavicular approach provides the most widespread surgical anaesthesia for the whole arm, whilst an infraclavicular approach often provides a pattern similar to the axillary approach.

An axillary approach provides good surgical anaesthesia for the elbow, forearm, and hand and also cutaneous anaesthesia of the inner upper arm including the medial cutaneous nerve of arm and intercostobrachial nerve. The axillary approach to the brachial plexus is considered the safest of the four approaches because of reduced risk to surrounding structures such as the risk of phrenic nerve blockade and/or pneumothorax, but the general risks of accidental intravascular and intraneural injection still exists.

## 4. Axillary Brachial Plexus Block

### 4.1. Indications

- surgical anaesthesia for elbow, forearm, and hand procedures,
- cutaneous anesthesia for superficial procedures of the inner arm, for example, brachiobasilic fistula formation,
- chronic pain treatment.

### 4.2. Techniques of Axillary Block

1. peripheral nerve stimulation,
2. ultrasound guided.

**(1) Peripheral Nerve Stimulation**

The use of a nerve stimulator for peripheral nerve blockade provided a definite advantage over traditional paraesthesia or transarterial techniques of the 1980s and became the most favoured modality used for peripheral block performance until the advent of ultrasound guidance. A multi-injection technique using a nerve stimulator was found to be associated with a higher success rate [5], as traditional single-injection approaches were limited by lack of circumferential spread of local anaesthetic due to the presence of septa within the axillary sheath, limiting the spread of local anaesthetic [6].

**(2) Ultrasound Guided**

In 1981, Abramowitz and Cohen described the first use of Doppler ultrasound to identify the axillary artery, thereby aiding the performance of axillary plexus block for upper limb surgery [7]. But it was the use of B-mode ultrasound in 1989 for axillary block performance that heralded the era of ultrasound-guided peripheral nerve block [8]. With the refinement of ultrasound technology and ultrasound-guided block techniques, it is gradually replacing nerve stimulator-based techniques. Ultrasonographic visualisation of target nerve, needle, and local anaesthetic injectate spread has been associated with improved block success rates [9–11], decreased block onset times [9–13], and a decrease in the local anaesthetic dose needed for successful nerve block [14–16].

### 4.3. Performance of Axillary Block under Ultrasound Guidance

**Figure 2:**
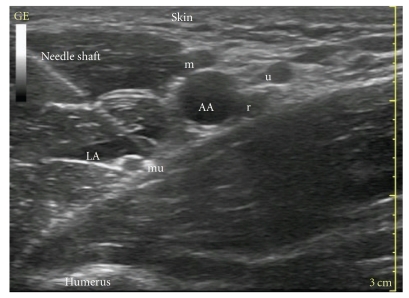
Ultrasound scan of axilla. AA: axillary artery, LA: local anaesthetics, r: radial nerve, mu: musculocutaneous nerve, m: median nerve, and u: ulnar nerve. This is an in-plane approach, with the whole length of the needle shaft visible under ultrasound.

The arm is abducted to 90 degrees and the elbow flexed to 90 degrees. The axilla is prepared aseptically and a high-frequency linear probe scans in a transverse plane at the lateral border of pectoralis major muscle. The pulsating axillary artery is visualized, and the transducer moved to locate the individual nerves around the artery. Easing the pressure off the transducer usually reveals the position of the axillary vein. The nerves at this level have a honeycomb appearance, but their locations relative to the artery are variable. The median nerve usually lies around 9–12 o'clock position, the ulnar nerve often in the corresponding 2 o'clock position, and radial at the 5 o'clock position in relation to the artery [17] (Figure 2). The musculocutaneous nerve usually lies in the plane between the biceps and coracobrachialis muscles or in the body of coracobrachialis and has a flattened appearance with a bright border and often a black, hypoechoic core. As we scan up and down the arm, the musculocutaneous nerve appears to glide in the fascial plane, either moving towards the artery as we scan proximally or away from the artery as we scan distally down the arm. The radial nerve, which lies deeper, below and medial to the artery is the one most difficult to visualise with ultrasound. It is important to exclude postcystic ultrasonographic enhancement beneath the artery, with which the radial nerve is most often confused. Various measures have been tried to obviate this clinical problem including the use of a peripheral nerve stimulator, scanning the radial nerve proximally, beginning from the radial groove on the humerus and tracking proximally into the axilla, and finally employing blind injection of local anesthetic at the 5 o'clock position in relation to the artery. Injection of local anesthetic in a “horse-shoe” pattern underneath the artery, with the needle tip at 5 o'clock position, in our experience, effectively blocks the radial nerve in most cases. A similar “donut” technique has been described by Imasogie et al. [18], where the authors achieved successful block of the median, ulnar, and radial nerves by circumferential deposition of local anaesthetic around the axillary artery, instead of targeting them individually.

**Figure 3:**
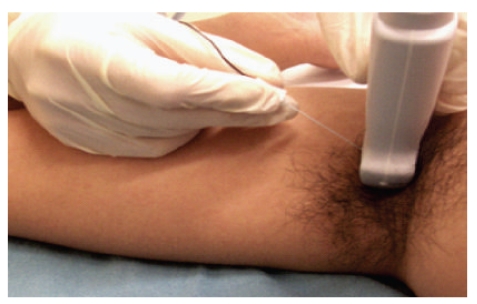
Ultrasound-guided axillary brachial plexus block. This is an example of an out-of-plane approach of the needle with respect to the probe.

After positioning of the probe, we recommend infiltration of local anaesthetic distal to the probe, subcutaneously, to cover the injection site and to block the intercostobrachial nerve. A short-bevelled 5 cm needle can then be inserted either in-plane or out-of-plane (Figure 3) relative to the probe, towards the four nerves and each blocked individually. The in-plane approach to axillary block [11] involves the insertion of the needle along the long axis of the probe, keeping the entire length of the needle in view during the procedure. The out-of-plane approach [19], in contrast, involves insertion of the needle along the short axis of the probe, and hydrolocation of the needle tip may be necessary to confirm the position of the tip of the needle. In terms of safety, the in-plane approach offers better visualisation of the needle tip [20], but the out-of-plane approach to axillary block has been shown to be more comfortable of the two, for the patient, in a recent study [21]. After careful positioning of needle tip, gentle negative aspiration, and an asymptomatic initial 0.5–1 mL perineural injection, further local anaesthetic is injected in 2 mL aliquots to surround each nerve.

### 4.4. Choice of Local Anaesthetic

The choice of local anaesthetic is determined by the duration of sensory analgesia desired. Lidocaine 1.5–2% with adrenaline 5 mcg·mL^−1^and Mepivacaine 1% provide effective nerve blockade for 2.5–3 hours and are ideal for shorter duration procedures [22, 23]. For longer-duration procedures, it is possible to achieve sensory blockade for 9 hours with Ropivacaine 0.5% and 11 hours with Levobupivacaine 0.33% [24]. When used for surgical anaesthesia decreasing the concentration of both local anaesthetics further would lengthen the onset of block and increase the risk of inadequate blocks [25].

### 4.5. Volume of Local Anaesthetic

In the past, it was necessary to use large volumes of local anaesthetic to achieve acceptable success rates for peripheral regional anaesthetic techniques. Recent studies have shown that volume of local anaesthetic can be significantly reduced when axillary blocks are performed under ultrasound guidance [22, 23, 26]. An ED95-volume of 0.11 mL/mm^2^of Mepivacaine has been shown to effective for individual nerves of axillary block, which translates into 0.7–1 mL of local anaesthetic for individual nerves [24]. However, it should be noted that the anatomy of the axillary brachial plexus involves three additional cutaneous nerves (ICB, MCNA, and MCNF) with extensive distribution in the arm and forearm, and the use of such low volumes may risk inadequate block in the distribution of these nerves. Also, it is important to remember that these doses are “adequate” in the hands of very experienced regional anaesthetists, and using such low volumes while learning to perform blocks under ultrasound would reduce success rates and decrease confidence in this technique. It is recommended by the authors to use at least 4-5 mls of local anaesthetic for each nerve during axillary nerve block.

## 5. Conclusion

Axillary nerve block is a safe and effective regional anaesthetic technique suitable for a wide variety of procedures, for both inpatient and outpatient care [27–32]. Ultrasound guidance has allowed improved efficacy with smaller volumes of local anaesthetic. Direct visualisation of block performance and local anaesthetic injection, though inherently safer, does not completely eliminate the risk of intravascular and intraneural injection, and care should be continually exercised using standard safety precautions of slow, careful, fractionated injections to prevent and minimise the risks associated with the technique.
